# Prof. Dr. O. Yavuz Ataman

**DOI:** 10.3906/kim-2101-49

**Published:** 2021-02-17

**Authors:** Ahmet Emin EROĞLU*

**Affiliations:** 1 Department of Chemistry, İzmir Institute of Technology, İzmir Turkey

**Keywords:** Prof. Dr. O. Yavuz Ataman, obituary

## Abstract

Prof. Dr. O. Yavuz Ataman passed away on August 15, 2020, in Ankara. He was an academic as well as a social figure throughout his life. He had remarkable achievements in academy as a researcher, an educator, and an administrator. He was known for his unique approaches to the events in all aspects of life. With his beloved character, he was really special. With Prof. Ataman’s passing away, the chemistry and especially analytical chemistry community have lost a very special member.

## Prof. Dr. O. Yavuz Ataman

Our dear professor, Prof. Dr. O. Yavuz Ataman, passed away on August 15, 2020, in Ankara. It was a heavy loss for his family, friends, students, and colleagues, as well as for all people who have known him. Although it is never easy to get used to the idea that we will not see him again in his office, classrooms, laboratories, and meetings, I am sure that his wisdom will always be with us.

What one can say about a person who has become an academic as well as a social figure throughout his life, who has been known for his unique approaches to the events, his deep thoughts, his remarkable achievements, and above all his beloved character? There is a lot to write about every single virtue of Ataman, such as his studentship, teaching abilities, education strategies, academic quality, research capacities, his virtues as an advisor, an administrator, and his musical life. I will try to be as objective as I can in this writing, knowing that it is not totally possible for me to be so. It is hard to be objective about my dear advisor, who had supported me my whole life whenever I needed his help. Still, I will try my best to give a snapshot about him from my perspective, feeling sure that all the readers who have known Ataman will approve my words.

Prof. Ataman was born on January 21, 1947, in Ankara, as the third child of Fatma Meliha and Ahmet Muhtar couple. With their three children, Yavuz and his elder sisters Bilgin and Gül, the Ataman family members were very close to each other. Yavuz Ataman completed his elementary, middle, and high school education in Ankara. After receiving their respective undergraduate degrees, Yavuz and Gülay (Erkoç) got married and became the Atamans in 1972. Their daughter Demet was born in 1976. Demet and Giray Sadık got married in 2013 and their son Ege came into the world in 2014, making Ataman the “bilge adam (wise man)” grandfather of his well-beloved grandson. 

Ataman graduated from the Department of Chemistry, Faculty of Arts and Sciences, at Middle East Technical University in 1970. He received his PhD in 1975 under the supervision of Prof. Harry B. Mark, from the Department of Chemistry, University of Cincinnati (USA), with the title “Determination of Ammonia in Aqueous Solutions by Infrared Spectroscopy Following Preconcentration on Zeolite”. After receiving his PhD, he joined the Department of Chemistry, Faculty of Arts and Sciences, at Middle East Technical University. He worked in the same department for more than 40 years until his retirement. He had been to the Chemistry Department at Cincinnati University as a visiting professor between 1986 and 1988. In subsequent years, he worked as an instructor in summer courses and advanced his research studies in Harry B. Mark’s research laboratory. This continued until 2003, the year in which Prof. Mark passed away. He became Associated Professor of Analytical Chemistry in 1981 and Professor of Analytical Chemistry in 1988. 

It was unlikely not to feel that Ataman had grown in a very warm environment of a very warm family. Occasionally, he mentioned his family and how his mother would call her granddaughter Demet as “Gülü Babaannesinin” (a common remark of affection in Turkish which can literally be translated as “the Rose of her Grandmother”); with a special emphasis on the words. You would easily deduce from such anecdotes how Ataman had a natural “analytical gift”, enabling him to pay attention to fine details of life. He showed care about the details of everything he did, like in writing a short or a long note, an official letter, an email, an article, in reading a thesis, etc. Observing him while doing these was a valuable experience. You would learn from him how to write even a very short note. In addition to his individual nature, the warm family atmosphere, the social structure around him, his sincere personality, self-confidence, discipline in his professional life, and the music hobbies had made Ataman a very special person.

In an article that he wrote years ago, he described himself as a student, a teacher, a researcher, and an administrator and explained the link between these identities. I will use his descriptions in this writing; in addition, I will allow myself to add some of his other virtues that he did not mention. These include his diligence, humanistic behavior, joy, passion, bravery, and patience. However, above all, his brand was his unifying character. He was a master of smooth transitions and like a social glue among people. 

Thanks to his knowledge and a strong background in diverse fields, he was able to walk around many things that are not directly related, which one would not easily understand how to achieve a smooth transition among them. His natural talent, the environment he grew up, his self-improvement, and his feeling of being responsible towards everything around himself have enabled him to manage all of these. 

We can understand the reasons why Ataman was deeply respected and beloved by considering all parts of his personality. 

Based on the long chats we had with Ataman, I may say a few words about his life as a student. It was not difficult to understand that Ataman was a bright student. He believed that being a good teacher stemmed from being a good student. This must be the underlying reason for the enthusiasm he had for learning/teaching. His family environment must have largely contributed to this. His father was a high school French teacher who enriched himself as a music historian. Ataman’s passion for music came from his father, who wrote lyrics for many famous classical music from well-known composers. Almost all of us know the Turkish lyrics of a Mozart composition “Daha dün annemizin kollarında yaşarken.....”. Although, at first glance, music appeared to be just a hobby in his life, the truth was it was more than that; during his university years, he earned his pocket money making music. He played bass guitar and took a role as vocalist in the bands, first “Kare As” and then “TNT”. Many years ago, in a coffee shop in Cincinnati, USA, one of his friends (Ümit Bey) told me this; “Your supervisor Yavuz used to play the guitar and sing on the stage over there.” Music was an essential part of his whole life. Following the rhythm of his adolescent times, he played and sang rock music. He used to sing very well. I do not remember where and when I read the following story, if it is a myth or not, or if Ataman told it to me; Eric Clapton went to listen to Jimi Hendrix, but he left the concert after a very short time. Some people asked him outside “You left early without waiting for the concert to end, was Hendrix too bad?”. Clapton replied enviously or jealously, “No, he was too good.” Ataman was Jimi Hendrix for us, he was Eric Clapton, he was Jimmy Page. On every occasion, his students asked him to sing the song “Hey Joe”, which became more famous after Hendrix sang it. Ataman also sang it many times. Might he had sung it more than Hendrix? He mentioned, from time to time, that he had come across some students of his father who shared their memories about his father playing the violin as a break when students got bored with heavy French grammar in the class, then continuing the lesson. Ataman, like his father, although not in the classroom, played the guitar for his students and fed their souls with his songs.

In my opinion, Ataman’s most important quality was his teaching abilities. His teaching was so natural that it made you feel that you are in a mystic world; all influential scientists who worked on the subject had come together through his body and verbalized the context with easy-to-understand words. He was gifted in teaching. He was explaining the course topics in a way that makes you think that you could do the same right away. You understand how difficult it was when you come across the same situation. 

He taught Analytical Chemistry, Instrumental Analysis, and several elective courses. His lessons were as if “understanding came with a smile”. His enthusiasm was contagious right after he entered the classroom. Have you ever seen a university student who was sad about missing a lecture? I have. His teaching approach on the course topics, making the difficult topics as simple as possible, his extraordinary intuition in choosing the right examples to make the topics clearer, his smooth/readable hand writing on the blackboard… You would like to take a photograph of the blackboard which he decorated with his colored chalks. He had notes to remind himself of the topics, but he always used analogies from daily life to explain the topics in a simple way. He was a master of explaining the most difficult-to-understand topics in the easiest way. None of his characteristics seemed like he borrowed it from somewhere else; it was his own. He was so smooth in transitions from one subject to another, and was also so successful in decorating the topics with the best examples, to the extent that makes you wonder at the end “What just happened?”. Everybody who attended any of his lectures knows what I mean. I never got bored and lost focus in the classes he was teaching (It was very difficult for me to listen to the courses due to my inattentiveness in classes.). I think the secret to his teaching ability is his unique approach to knowledge. To be more clear, he always believed that anything that we came across might be helpful at some point and must not be considered unimportant; therefore, he stored the events and the related things in the synaptic networks created deep in his brain and brought them alive whenever necessary. As a result, his learning, teaching, and distribution of his thoughts through verbal and written communication were extraordinary.

His academic style was not limited to the classroom courses. You would notice Ataman having a chat with the surrounding students. Memories, anecdotes, proverbs would abound; everybody would chat about anything from deep academic problems to up-to-date topics. There would always remain something from these chats. The reason for this pleasant atmosphere was of course Ataman’s warm character. He would have a chat with a small child in the same manner and put a new thing into the memory of the child from that moment. With his well-spoken and humorous character, he would dive into interesting topics and always bring beauty to the surface. 

In his academic life, Ataman had worked on atomic and molecular spectrometry and taught many students. He was influential, especially in the field of atomic spectrometry in Turkey and in the world. He supervised 17 PhD and 43 MSc students, enabled 7 postdoctoral researchers to work and gain a vision in his research laboratories. He published around 80 scientific papers and wrote two books. Although he worked on spectrometry, he did not hesitate to deal with various scientific fields. He mentioned a principle that he taught to his students: “Sometimes, you write a project proposal, you constitute your team, you get the necessary equipment and the chemicals for your project, and then you try to finish the project. This approach is correct. Some other times, while already working on a research topic, you heard about a new formation of a team, you meet with new people, a new instrument is brought nearby, you get inspired from this new progress, you think of being a part of the new formation. Then, you realize that you have come up with a new idea that was not in your mind before. This approach is also correct. Therefore, you doubtlessly need to concentrate on your topic, but, at the same time, do not give up watching around.” Actually, he was talking about a type of collective working, which is imperative in today’s research approaches. “Sometimes, it is what it is. It is all that you can do with it. You must be in search, but, at the same time, appreciate what you have.” It is still valid, isn’t it?

Ataman put emphasis on administrative duties for which some other academics would not show an interest. He served as the chairman of the Chemistry Department and the dean of the Arts and Sciences Faculty, at Middle East Technical University. He was a member of Administrative Board of Society for Accreditation of Undergraduate Programs in Faculties of Arts and Sciences (FEDEK). FEDEK committees are composed of deans of science faculties from many universities in Turkey. These committees made progress on various subjects that are critical for academia. He also worked on the accreditation of the courses and the laboratories in the curricula of the Chemistry departments. He spent a good deal of time to update the course contents. He really put effort into the Chemistry Olympiads. He served as the chairman of the Ankara branch of the Turkish Chemical Society for more than 20 years. He was a member of a wide range of scientific societies and editorial/advisory boards of scientific journals. I was always impressed by his ability to manage his limited time.

Ataman tried hard to sort out the things he dealt with. He showed interest in not only the general and relatively big issues but also the so-called smaller problems. He did not abstain from the topics that were difficult to solve. He could manage to solve some of them, some of them remained unsolved. However, he made an improvement in almost all of them. 

Contrary to the prevalent approach of dividing sciences into “natural sciences” and “social sciences”, he insisted on the belief that these two broad scientific classes are closely related or must be related to each other and must be nourished from each other. You could see this reflected on his behavior on every duty that he realized.

He was one of the leading academics in the organization of many national and international conferences. For instance, Colloquium Spectroscopicum Internationale XXXI (CSI XXXI) was held in Ankara (Turkey) in 1999 under the chairmanship of Ataman. In addition, he played a very significant part in many scientific committees of important conferences such as National Spectroscopy Congress, IUPAC Congress, National Chemistry Conference, Aegean Analytical Chemistry Days, National Analytical Chemistry Congress, Black Sea Basin Conference on Analytical Chemistry and the like. These conferences had been the place for the young scientists to meet with and be inspired from influential scientists from different countries. He delicately accomplished all duties he was asked for. As a sensitive person, he dealt with everybody and everything he was responsible for. He polished the topics he had to. Due to his self-confidence on the very broad spectrum, he liked to work independently; but whenever necessary, he got help from anybody whose experience and knowledge had to be appreciated. He was the master of discussion sessions, complicated meetings, and many responsibilities that appeared difficult for many people. During presentations, talks, scientific discussions in the meetings where a different look was needed, Ataman was there to stimulate the occasion. This was the result of him being very knowledgeable on a very broad range of different issues. His research quality, international personality, personal relationships, and leadership, all together paved the way for becoming the unifying glue/cement of the meetings. This increased his workload, but he used to take it with pleasure, without complaints. Nobody would give such a responsibility to him; it would just naturally happen. In those meetings, Ataman was one of the wise men to be followed.

His social abilities were extraordinary. Due to his warm personality, he used to put forward positive things on all occasions. Whenever he had to, he criticized others’ work gently. Critiques that could make you feel humiliated when made by some other people would be very informative/beneficial when put into words by Ataman. One day, he was sharing information; another day, he was talking about a completely different and interesting subject like a social event or an artistic activity. No subject was out of his area of interest. I think this part of his character was one of the important ingredients of his teaching ability.

He was the advisor of every student without any discrimination. He made possible for many students to study in good universities abroad using his international personality. He demonstrated to write scientific texts both in Turkish and in English. He kept connections with his students even when they were abroad. His supervision was endless. He trusted his students, encouraged them to take responsibility, and to act independently. All of his students benefited from his supervision. His proofreading was incomparable; he was the best in decorating your texts with red color. He used to say “İyi bahçıvan zalim olurmuş (a good gardener needs to be cruel)”. The writing and scientific quality of the texts he revised would be enhanced enormously. He was never just a supervisor. He supported all students at critical moments in their lives. Irrespective of the department/university of the students, Ataman tried to help all of them gain quality and sophisticated experience not only in their academic career but also their entire lives. The Atamans had a ritual for student graduations. Mrs. and Mr. Ataman would invite all group members to their house and treated all of their guests with enormous attention. I understand the value of these meetings better now. These nights always ended with Ataman singing songs and playing the guitar. “Hey, Joe, where you goin’ with that gun in your hand?” 

Ataman was the social glue of every occasion he belonged to. He was really special.

With Ataman’s passing away, the chemistry and especially analytical chemistry community have lost a very special member. Nowadays, AAS flames are extinguished, ICPs are cold, pH indicators are colorless, and blackboards are erased. Atoms are not walking around. Do not worry Hocam; they’ll be fine in time. 

Hey Joe, where you goin’ with all that memories in your soul?

Hey Joe, where you gonna run to now, where you gonna run to?

